# The Association between Access to Public Transportation and Self-Reported Active Commuting

**DOI:** 10.3390/ijerph111212632

**Published:** 2014-12-05

**Authors:** Sune Djurhuus, Henning S. Hansen, Mette Aadahl, Charlotte Glümer

**Affiliations:** 1 Research Centre for Prevention and Health, The Capital Region of Denmark, Glostrup University Hospital, Nordre Ringvej 57, Section 84–85, DK-2600 Glostrup, Denmark; E-Mails: Sune.djurhuus@regionh.dk (S.D.); Mette.Aadahl@regionh.dk (M.A.); 2 Department of Planning, Alborg University Copenhagen, A.C. Meyers Vænge 15, DK-2450 Copenhagen, Denmark; E-Mail: hsh@plan.aau.dk; 3 Department of Health Sciences and Technology, Aalborg University, Frederik Bayers Vej 7D2, DK-9220 Aalborg, Denmark

**Keywords:** active commuting, GIS, multi-level regression, travel planner data, origin-destination

## Abstract

Active commuting provides routine-based regular physical activity which can reduce the risk of chronic diseases. Using public transportation involves some walking or cycling to a transit stop, transfers and a walk to the end location and users of public transportation have been found to accumulate more moderate physical activity than non-users. Understanding how public transportation characteristics are associated with active transportation is thus important from a public health perspective. This study examines the associations between objective measures of access to public transportation and self-reported active commuting. Self-reported time spent either walking or cycling commuting each day and the distance to workplace were obtained for adults aged 16 to 65 in the Danish National Health Survey 2010 (n = 28,928). Access to public transportation measures were computed by combining GIS-based road network distances from home address to public transit stops an integrating their service level. Multilevel logistic regression was used to examine the association between access to public transportation measures and active commuting. Distance to bus stop, density of bus stops, and number of transport modes were all positively associated with being an active commuter and with meeting recommendations of physical activity. No significant association was found between bus services at the nearest stop and active commuting. The results highlight the importance of including detailed measurements of access to public transit in order to identify the characteristics that facilitate the use of public transportation and active commuting.

## 1. Introduction

There is convincing evidence that engaging in regular moderate-to-vigorous physical activity reduces the risks of obesity [1,2], cardiovascular diseases [3], diabetes [4] and premature death [5]. Active commuting is receiving increased attention in this context because its routine-based nature provides regular physical activity during the week. Active commuting can be performed walking or cycling all the way to work or study, as part of a multimodal trip (e.g., walking or cycling in combination with using public transportation), that involves some walking or cycling to a transit stop, transfers and a walk to the end location, or driving in a car some of the way in combination with walking, cycling or public transportation [6]. Evidence has shown that active commuters are more likely to reach the 2010 WHO [7] recommendations of 150 min of moderate intensity physical activity (MPA) per week [8,9]. Public transportation commuters have been found to accumulate significantly more MPA per day (5–10 min) [10] compared to non-public transportation users. Those who commute solely by active modes or active commuting in combination with public transportation or car have been found to be significantly more active than those who use motorized modes of transport [11]. In addition, studies with pedometer measurements have found that public transportation commuters walk significantly more steps per day than car commuters [8,9]. In a public health perspective, active commuting by public transportation has been found to be associated with a significantly lower risk of being overweight [4] or obese (men only) [2] and active commuting has been found to have a protective effect on cardiovascular outcomes [3] and may have a positive effect on diabetes prevention [4,12,13]. Studying local public transit characteristics that facilitate the use of public transportation thus addresses key information needed to promote active transportation and resulting health benefits.

Previous studies have established that the physical environment plays an important role in active commuting and associations have been found between active commuting and objective measures of the built environment, walking and biking facilities, street connectivity and proximity of destinations [14,15,16]. Furthermore, the commute distance has been found to be negatively associated with active commuting [17]. Several studies of access to public transportation and its association with active commuting have characterised access to public transportation by distance to the nearest stop [15,18,19,20,21] or density of stops within variable distances [22,23,24,25,26]. A few of these studies have found that distance to transit [15,19] is negatively associated and that density of stops [22,24,25,26] is positively associated with active commuting. However, additional relevant measures of public transportation access such as service frequency, route variation and meaningful destinations [27], have received much less attention. A few studies of the association between active commuting and public transportation include service frequency at the nearest stop [15,21] or available bus routes [15]. Only Dalton *et al.* [15] find a positive association between nearest bus stop service frequency and active commuting.

The aim of the present study was to examine the associations between a range of different objective measures of access to public transportation and self-reported active commuting in The Capital Region of Denmark. Transport service characteristics (service frequency, routes and transport modes) at the nearest, the best connected stop and all stops within walking distance were included in the objective measures. We further investigated if the associations were modified by the commute distance, age and gender.

## 2. Experimental Section

### 2.1. Study Population and Data Sources

The study population comprised a subsample of the Danish National Health Survey 2010. The study design has been described in detail previously [28]. This paper focused on respondents living in The Capital Region of Denmark. The region includes urban, suburban and rural districts. A random sample of 95,150 was selected from the total population above 16 years of age living in the Capital Region of Denmark (1,355,000). Data was collected from February to April 2010 and the response rate was 52.3%. Selected respondents for this study were 16 to 64-year-olds either working or under education, with a commute distance of up to 200 km, living on the main island of Zealand who provided valid answers on commuting. This reduced the study sample to 28,928 respondents. The respondents´ home addresses were subsequently geocoded using the official Danish Address Register from the Danish Geodata Agency. The survey contained a wide range of self-reported questions on health status and health behaviour, as well as socio-demographic characteristics, including distance to and time spent walking or cycling to work or study each day. Individuals were invited to participate in the survey by answering an enclosed paper questionnaire and returning it by the mail, or online. In addition to the questionnaire, register-based data on individual respondents’ age, gender, education and income were obtained from Statistics Denmark.

Two main geographical data sources were used in the study. Data on transit stop location, transport mode, routes and time schedules were obtained from www.rejseplanen.dk, the official search engine for information about public transportation in Denmark. The Capital Region of Denmark has four major transport modes: bus, train, s-train (light-rail) and metro. The data covered February to April 2010 in accordance with the time period of the questionnaire. A road network containing all road types and walking and cycling paths was obtained from the Danish Geodata Agency (Kort10) to conduct the walking/cycling distance measurements. Roads where walking or cycling was not permitted (e.g., motorways, highways) were excluded from the dataset.

The Health Survey was reported to the Danish Data Protection Agency. Approval from the regional Committee on Health Research Ethics was not necessary as no human biological material was included in the data collection.

### 2.2. Definition of Variables Used in the Study

#### 2.2.1. Active Commuting

The outcome variable was based on self-reported time spent walking or cycling to work every day (hours, minutes) [29]. The variable was dichotomized into being an active commuter “yes” or “no”, with a cut-off value of 5 min spent on active commuting per day and meeting recommended levels of physical activity (≥30 min) per day “yes” or “no”.

#### 2.2.2. Objective Measures of Access to Public Transportation

The objective measures of access to public transportation were determined by combining the geographical location of the home address of each participant in the study population, road networks from the Danish Geodata agency and the geographic location of transit stops and their service level. Network distances from each respondent’s home address to the nearest stop (bus, metro, s-train, train) and to all stops within 1 km walking and 3 km cycling distance were calculated using origin—destination matrices in the Network Analyst application of ESRI ArcGIS 10.1. (Environmental Systems Research Institute, Redlands, CA, USA).

#### 2.2.3. Distance to and Density of Public Transportation Stops

The access to public transportation was constructed as distance and density measures. The distance to the nearest bus stop and train station was treated both as a continuous and a categorical variable. The distance to metro or S-train was not linearly related to the outcome and was therefore not treated as continuous measures. Distance and density measures have often been categorised into distances of 400 m [15,26,30] and 800 m [15,19,26] to reflect the distances people are willing to walk to a bus stop or a train station. The distances have been challenged by a number of studies using travel diary data and distance-decay functions [31,32]. Millward *et al.* [32] found a peak in walking to bus stops at distances between 200 m and 400 m and that most walks to a number of destinations was covered within 1 km. Krygsmann *et al.* [31] found that 50 % of people were willing to walk 550 m and cycle 1.8 km to access a station. As 76.6% of the respondents resided within 400 m of a bus stop, it was decided to categorise respondents into residing right next to a bus stop (0–200 m), residing within immediate walking distance (200–400 m), residing within a medium long walking distance (400–800 m) and having a long walking distance to a bus stop (>800 m). The other transport modes had much lower access coverage, which is in line with the longer distance used for accessing train stations. The categorization was determined by living at a closer or further location for walking distance (0–500m and 501–1000 m), cycling distance (1001–3000 m) and more than 3000 m from a station. The cycling distance to the train, S-train and metro was inspired by a travel survey performed in the Capital Region of Denmark in 2006–2007 [33], showing that the average cycling distance for any purpose was 3 km cycling. Density of bus stops was defined as the number of bus stops within 1 km walking distance from the home address. The density was divided into four categories based on the density distribution: 0–5, 6–10, 11–15 and >15 stops. As an alternative density measure, an index of transport modes reachable within 1 km and 3 km network distance from home address was created. The index with values from 0 to 4 was defined as Equation (1):

(1) TMI = BUS(0,1) + Metro(0,1) + S−Train(0,1) + Train(0,1)

No stop within 1 km for each mode was taken to equal 0, and 1 if a stop was present.

#### 2.2.4. Service Level of Public Transportation

Distinct active bus routes at the nearest bus stop and service frequency (number of departures) at the nearest and the “best” stop was extracted from www.Rejseplanen.dk. The time period covered were the morning rush hour (7:00 a.m. to 8:00 a.m.). The “best” connected stop was defined as the stop with the highest service frequency within 1 km walking distance from the individual home address. In addition to the distance density measures, a measure of unique bus routes reachable within 1 km walking distance was created. Distinct bus routes at the nearest stop were divided into three categories and unique bus routes within 1 km walking distance and the frequency of services was divided into four categories based on the distribution of data. Inspired by Kamada *et al.* [21], two bus convenience measures were created combining the distance and service frequency at the nearest stop and distance and service frequency at the “best” connected stop, as shown in Table 1.

**Table 1:** Categorization of the bus convenience based on bus frequency and distance to a bus stop with 4 indicating the highest bus convenience.

Distance to Bus Stop	Bus Frequency			
	High	Medium-High	Medium-Low	Low
Close	4	4	3	2
Medium close	4	4	3	2
Medium far	3	3	1	1
Far	2	2	1	1

#### 2.2.5. Socio-Demographic Covariates

The individual socio-demographic covariates were register-based age, gender [34] and education level [35]. Four classes of education level were defined: primary or secondary school, vocational education, bachelor degree or equivalent, and master’s or PhD degree.

#### 2.2.6. Contextual Covariates

Median income level, population density and street connectivity were grouped by parishes, the smallest administrative units in Denmark. Median income level and population were based on individual data from central registers [34,36]. Street connectivity was defined by the gamma index γ = l/(3(n – 2), where n equals the intersections [37].

#### 2.2.7. Statistical Analyses

Prior to running the analyses, a pair-wise correlation matrix was constructed to identify variables that were highly correlated. Highly correlated variables were defined as having a Pearson’s correlation coefficient of >0.55 [15]. The results were used to evaluate the risk of multicollinearity in the multilevel models. We used SAS version 9.3 (SAS Institute, Inc., Cary, NC, USA) to perform the multilevel regression analyses (GLIMMIX procedure) to investigate which of the access to public transportation measures was associated with being an active commuter and meeting recommended levels of physical activity. A two-level model was fitted with individuals (Level 1, n = 28,928) nested within parishes (Level 2, n = 223).

Two empty models (active commuter and meeting recommendations of physical activity with no fixed effects) were estimated to detect whether there was a contextual dimension to (1) being an active commuter and to (2) meeting recommended levels of physical activity. The contextual dimension was estimated by calculating the Intra Class Correlation Coefficient (ICC). A 3-step modeling strategy was used and the ICC was calculated for each model: (1) the primary determinant was included in the model; (2) the individual level covariates were included to examine whether the between-parishes variance was attributable to a compositional effect. Individual age, gender and education was included in all of the models; (3) the parish level contextual covariates were included to see if the remaining between-parish variance could be explained by contextual factors. Furthermore to see if the associations differed among subgroups, it was examined if there was a significant interaction with distance to work or study expressed by having commute distances of ≤5 km, 5–10 km, 10–20 km and ≥20 km, with age defined by three groups (16–29, 30–45, 46–64 years) and gender. Distance has been shown to be a strong predictor of travel mode choice [15,38,39]. The used distance categories are an aggregation of the categories used by Scheiner [39] and was inspired by the Danish National Travel Survey [40] that showed different commute patterns within the four distance groups. Travel mode to work has been found to differ with age and gender [4]. The three age groups were inspired by Laverty *et al.* [4] although they use four age categories. Values of *p* < 0.05 were considered statistically significant. If an interaction was present, the odds of being an active commuter when belonging to a given distance or age category were calculated based on the full model.

## 3. Results and Discussion

### 3.1. Results

#### 3.1.1. Demographics and Public Transportation Access

Of the study population, 72.9% reported active commuting each day and 50.6% met recommended levels of physical activity (moderate intensity physical activity) from active commuting alone (Table 2). Respondents with a vocational education had the lowest proportion of active commuters (63.5%). Women had a higher proportion of active commuters than men and the proportion of active commuters decreased with increasing commute distance and age.

**Table 2:** Descriptive statistics of study population demographics and distances to work by subgroups of active commuters (≥5 min/day) (yes/no) and meeting recommendations of physical activity (yes/no).

	Total	Active Commuter (≥5 min/day)		Meeting Recommended Levels of Physical Activity (≥30 min/day)	
		Yes	No	Yes	No
	n (%)	n (%)	n (%)	n (%)	n (%)
**Total population**	28,928 (100)	21,094 (72.9)	7834 (27.1)	14,629 (50.6)	14,299 (49.4)
**Age ^a^**	40.9 (13.1)	39.7 (13.5)	44.3 (11.2)	39.3 (13.7)	42.6 (12.2)
**Age groups (6 missing)**					
16–29 years	6538 (22.6)	5724 (87.5)	814 (12.5)	4245 (64.9)	2293 (35.1)
30–45 years	10,782 (37.3)	7507 (69.6)	3275 (30.4)	5056 (46.9)	5726 (53.1)
46–64 years	11,604 (40.1)	7860 (67.7)	3744 (32.3)	5327 (45.9)	6277 (54.1)
**Gender (6 missing)**					
Male	12,624 (43.6)	8518 (67.5)	4106 (32.5)	5709 (45.2)	6915 (54.8)
Female	16,300 (56.3)	12,573 (77.1)	3727 (22.9)	8919 (54.7)	7381 (45.3)
**Education (438 missing)**					
Primary or secondary school	8150 (28.2)	6434 (78.9)	1716 (21.1)	4608 (56.5)	3542 (43.5)
Vocational education	7742 (26.8)	4920 (63.5)	2822 (36.5)	3273 (42.3)	4469 (57.7)
Academy or bachelor degree	7898 (27.3)	5822 (73.7)	2076 (26.3)	3992 (50.5)	3906 (49.5)
Master or PhD degree	4723 (16.3)	3593 (76.1)	1130 (23.9)	2501 (53.0)	2222 (47.0)
**Commute distance**					
≤5 km	9237 (31.9)	7957 (86.1)	1280 (13.9)	5731 (62.0)	3506 (38.0)
5–10 km	6676 (23.1)	5117 (76.6)	1559 (23.4)	3995 (59.8)	2681 (40.2)
10– 20 km	6516 (22.5)	4265 (65.5)	2251 (34.5)	2730 (41.9)	3786 (58.1)
>20 km	6499 (22.5)	3755 (57.8)	2744 (42.2)	2173 (33.4)	4326 (66.6)

**^a^** Age is expressed by mean and standard deviation.

The mean commute distance was 14.6 km (SD = 15.9), see Table 3. Active commuters reported shorter commute distances (12.7 km) than non-active commuters (19.6 km). Mean individual distance to the nearest bus stop was 300 m, whereas the mean distance to train and S-train was approximately 4 km and 13.3 km to the nearest metro stop. Active commuters had on average shorter mean distances to nearest train, s-train and metro stop than non-active commuters.

**Table 3:** Distance to the different public transportation modes in the population by subgroups of active commuters (≥5 min/day) (yes/no) and meeting recommendations of physical activity (yes/no).

km	Total	Active Commuting (≥5 min/day)		Meeting Recommended Levels of Physical Activity (≥30 min/day)	
		Yes	No	Yes	No
	Mean (SD)	Mean (SD)	Mean (SD)	Mean (SD)	Mean (SD)
Distance to work or education	14.6 (15.9)	12.7 (14.8)	19.6 (17.6)	11.8 (14.0)	17.1 (17.2)
Distance to a bus stop	0.3 (0.2)	0.3 (0.2)	0.4 (0.3)	0.3 (0.2)	0.3 (0.3)
Distance to a train station	4.2 (3.5)	4.0 (3.3)	4.8 (4.0)	3.8 (3.1)	4.6 (3.8)
Distance to a S-train station	4.1 (5.8)	3.7 (5.4)	5.3 (6.6)	3.3 (5.0)	5.0 (6.4)
Distance to a metro stop	13.3 (14.2)	11.6 (13.3)	17.9 (15.5)	10.1 (12.4)	16.6 (15.1)

#### 3.1.2. Association between Distance to Public Transportation and Active Commuting

The unadjusted models showed that distance to nearest bus stop or train station was negatively associated with being an active commuter (Table 4).

**Table 4:** Crude and adjusted associations (OR) between objective distance measures to public transportation and being an active commuter and meeting recommended levels of physical activity. Between neighbourhood variation is expressed by Intra-class correlation coefficient (ICC). Significant associations are highlighted in bold text.

Distance Measure	Active Commuter (≥5 min/day)		Meeting Recommended Levels of Physical Activity (≥30 min/day)	
	Model 1: Crude	Model 3: Fully Adjusted Model	Model 1: Crude	Model 3: Fully Adjusted Model
	OR (CI)	OR (CI) ^b^	OR (CI)	OR (CI) ^b^
**Distance to bus stop (km)**	**0.71 (0.63–0.80)**	**0.76 (0.67–0.85)**	**0.8 (0.71–0.90)**	**0.86 (0.76–0.96)**
*P-value ^a^*	*<0.0001*	*<0.0001*	*0.0002*	*0.0099*
*ICC*	*12.6*	*2.1*	*11.9*	*2.1*
**Distance to bus stop (m)**				
Close (≤200)	1.00	1.00	1.00	1.00
Moderate Close (201–400)	1.00 (0.94–1.07)	1.02 (0.95–1.09)	1.01 (0.95–1.07)	1.02 (0.96–1.08)
Moderate Far(401–800)	**0.88 (0.82–0.96)**	**0.92 (0.85–1.00)**	0.94 (0.87–1.01)	0.98 (0.91–1.05)
Far (>800)	**0.68 (0.58–0.80)**	**0.73 (0.62–0.86)**	**0.75 (0.63–0.88)**	**0.79 (0.67–0.94)**
*P-value ^a^*	*<0.0001*	*<0.0001*	*0.0010*	*0.0183*
*ICC*	*12.8*	*2.1*	*12.0*	*2.1*
**Distance to train station (km)**	**0.93 (0.91–0.94)**	**0.97 (0.96–0.98)**	**0.94 (0.93–0.96)**	**0.98 (0.97–0.99)**
*P-value ^a^*	*<0.0001*	*<0.0001*	*<0.0001*	*0.001*
*ICC*	*11.3*	*2.1*	*10.9*	*2.1*
**Distance to train station (m)**				
Close (0–500)	1.00	1.00	1.00	1.00
Medium Close (501–1000)	0.92 (0.76–1.12)	0.97 (0.79–1.18)	1.08 (0.90–1.29)	1.13 (0.95–1.35)
Medium Far (1001–3000)	0.84 (0.69 – 1.02)	0.86 (0.71–1.03)	1.03 (0.87–1.23)	1.06 (0.90–1.26)
Far (>3000)	**0.65 (0.52–0.80)**	**0.75 (0.62–0.91)**	0.88 (0.72–1.07)	0.99 (0.83–1.18)
*P-value ^a^*	*<0.0001*	*0.0002*	*0.0101*	*0.1254*
*ICC*	*12.8*	*2.1*	*12.2*	*2.1*
**Distance to S-train station (m)**				
Close (0–500)	1.00	1.00	1.00	1.00
Medium Close (501–1000)	0.99 (0.87–1.12)	1.03 (0.90–1.17)	1.02 (0.91–1.13)	1.03 (0.93–1.15)
Medium Far (1001–3000)	**0.79 (0.70–0.90)**	0.89 (0.78–1.00)	0.90 (0.80–1.00)	0.96 (0.86–1.07)
Far (>3000)	**0.53 (0.44–0.62)**	**0.81 (0.69–0.94)**	**0.64 (0.55–0.75)**	**0.87 (0.76–1.00)**
*P-value^a^*	*<0.0001*	*0.0002*	*0.0017*	*0.0260*
*ICC*	*9.6*	*2.0*	*9.3*	*1.9*
**Distance to metro stop (m)**				
Close (0–500)	1.00	1.00	1.00	1.00
Medium Close (501–1000)	0.83 (0.66–1.04)	0.86 (0.68–1.08)	1.03 (0.87–1.21)	1.04 (0.88–1.22)
Medium Far (1001–3000)	**0.66 (0.52–0.84)**	**0.78 (0.63–0.98)**	0.93 (0.77–1.12)	1.05 (0.89–1.24)
Far (>3000)	**0.27 (0.21–0.35)**	**0.56 (0.45–0.71)**	**0.42 (0.35–0.51)**	**0.74 (0.61–0.88)**
*P-value ^a^*	*<0.0001*	*<0.0001*	*0.0017*	*<0.0001*
*ICC*	*5.2*	*1.6*	*5.8*	*1.7*

**^a^** *P*-value from type III test of the association. **^b^** All models adjusted for neighbourhood confounders population density, median income, street connectivity and individual confounders. Bus distance adjusted for age, gender, education, bus routes and bus frequency. Train, S-train and metro adjusted for age, gender, education and distance to bus.

After adjusting for potential confounders, greater distance to a bus stop and a train station was associated with significantly lower odds of being an active commuter as well as with meeting recommended levels of physical activity. Residing >400 m from a bus stop was associated with significantly lower odds of being an active commuter compared to residing within 400 m, and residing >800 m from a bus stop was associated with significantly lower odds of being an active commuter compared to residing within 800 m. For trains, S-trains and metro there was a similar dose-response trend, as greater distance to a station was associated with lower odds of being an active commuter. For trains and S-trains, there was only a significant difference in the association for those residing >3 km from a train or S-train station compared to residing within 500 m. The adjusted models for meeting recommendations of physical activity showed that for trains, metro and S-trains, there was only a significant difference in the association for those residing >3 km compared to residing within 500 m.

#### 3.1.3. Association between Density and Service of Public Transportation and Active Commuting

The categorised density and service measures and their association with active commuting are shown in Table 5 and Table 6. For the adjusted models, density of bus stops, bus routes within 1 km and number of transport modes within walking and cycling distance were all positively associated with being an active commuter.

**Table 5:** Crude and adjusted associations (OR) between objective density measures of public transportation and being an active commuter and meeting recommended levels of physical activity. Between neighbourhood variation is expressed by Intra-class correlation coefficient (ICC). Significant associations are highlighted in bold text.

Density Measure	Active Commuter (≥5 min/day)		Meeting Recommended Levels of Physical Activity (≥30 min/day)	
	Model 1: Crude	Model 3: Fully Adjusted Model	Model 1: Crude	Model 3: Fully Adjusted Model
	OR (CI)	OR (CI) ^b^	OR (CI)	OR (CI) ^b^
**Density of bus stops**				
Low (0–5)	1.00	1.00	1.00	1.00
Medium low (6–10)	**1.29 (1.20–1.39)**	**1.25 (1.16–1.34)**	**1.19 (1.11–1.28)**	**1.16 (1.08–1.25)**
Medium high (11–15)	**1.56 (1.42–1.71)**	**1.32 (1.20–1.45)**	**1.38 (1.26–1.51)**	**1.22 (1.12–1.34)**
High (>15)	**2.42 (2.12–2.76)**	**1.52 (1.32–1.75)**	**1.64 (1.46–1.85)**	**1.22 (1.08–1.38)**
*P-value* ^a^	*<0.0001*	*<0.0001*	*<0.0001*	*<0.0001*
*ICC*	*6.4*	*1.8*	*8.4*	*1.9*
**Bus routes at stops within 1 km**				
Low (0–2)	1.00	1.00	1.00	1.00
Medium low (3–4)	**1.17 (1.08–1.26)**	**1.14 (1.05–1.23)**	**1.10 (1.02–1.19)**	**1.09 (1.01–1.17)**
Medium High(5–6)	**1.49 (1.34–1.65)**	**1.27 (1.14–1.41)**	**1.30 (1.18–1.43)**	**1.18 (1.07–1.29)**
High (>6)	**1.75 (1.56–1.96)**	**1.31 (1.16–1.48)**	**1.32 (1.19–1.46)**	**1.09 (0.98–1.22)**
*P-value* ^a^	*<0.0001*	*<0.0001*	*<0.0001*	*0.0082*
*ICC*	*8.1*	*1.8*	*9.8*	*2.0*
**TMI 1 km**				
0 ^c^	**0.67 (0.53–0.83)**	**0.67 (0.54–0.85)**	**0.77 (0.60–0.98)**	**0.78 (0.61–0.99)**
1	1.00	1.00	1.00	1.00
2	**1.29 (1.20–1.40)**	**1.19 (1.11–1.29)**	**1.18 (1.10–1.27)**	**1.12 (1.04–1.19)**
3	**1.53 (1.30–1.79)**	**1.35 (1.16–1.56)**	**1.14 (1.00–1.30)**	**1.07 (0.94–1.20)**
*P-value* ^a^	*<0.0001*	*<0.0001*	*<0.0001*	*0.0018*
*ICC*	*10.7*	*1.9*	*11.1*	*2.0*
**TMI 3 km**				
1^ c^	1.00	1.00	1.00	1.00
2	**1.35 (1.21–1.51)**	**1.19 (1.07–1.33)**	**1.29 (1.15–1.45)**	**1.16 (1.04–1.29)**
3	**1.85 (1.61–2.12)**	**1.42 (1.24–1.62)**	**1.70 (1.49–1.95)**	**1.38 (1.21–1.57)**
4	**4.30 (3.57–5.18)**	**1.87 (1.53–2.28)**	**2.79 (2.35–3.31)**	**1.44 (1.21–1.71)**
*P-value* ^a^	*<0.0001*	*<0.0001*	*<0.0001*	*<0.0001*
*ICC*	*4.9*	*1.7*	*6.0*	*1.7*

**^a^** *P*-value from type III test of the association. **^b^** All models adjusted for neighbourhood confounders population density, median income, street connectivity. Bus distance adjusted for individual confounders age, gender, education, bus routes and bus frequency. Train, S-train and metro adjusted for individual confounders age, gender, education and distance to bus. **^c^** The number represents number of transport modes within walking(1 km) and cycling distance (3 km).

No significant associations were found between bus service measures at the nearest stop (routes and service frequency) and being an active commuter. A higher bus convenience (combined distance with service frequency) at the nearest stop was associated with significantly higher odds of being an active commuter except for the medium-low category. No significant association was found between the bus convenience at the “best” stop and being an active commuter. In the adjusted models for meeting recommended levels of physical activity, density of bus stops, the bus frequency at the “best” stop and transport modes within cycling distance were positively associated with meeting recommendation of physical activity.

**Table 6:** Crude and adjusted associations (OR) between objective measures of public transportation services and being an active commuter and meeting recommended levels of physical activity. Between neighbourhood variation is expressed by Intra-class correlation coefficient (ICC). Significant associations are highlighted in bold text.

Bus Service Measure	Active Commuter (≥5 min/Day)		Meeting Recommended Levels of Physical Activity (≥30 min/Day)	
	Model 1: Crude	Model 3: Fully Adjusted Model	Model 1: Crude	Model 3: Fully Adjusted Model
	OR (CI)	OR (CI)	OR (CI)	OR (CI)
Bus routes at nearest stop				
Low (≤1)	1.00	1.00	1.00	1.00
Medium (2)	1.00 (0.93–1.07)	0.98 (0.91–1.05)	1.00 (0.93–1.06)	1.00 (0.93–1.07)
High (>2)	1.03 (0.95–1.12)	0.97 (0.88–1.07)	0.94 (0.87–1.01)	0.92 (0.84–1.01)
*P-value* ^a^	*0.7272*	*0.7919*	*0.1362*	*0.1372*
*ICC*	*13.7*	*2.0*	*12.7*	*2.1*
Frequency of bus service at nearest stop				
Low (0–2)	1.00	1.00	1.00	1.00
Medium-low (3–6)	**0.90 (0.83–0.98)**	0.92 (0.85–1.01)	**0.91 (0.84–0.99)**	0.95 (0.88–1.03)
Medium-high (7–15)	1.02 (0.93–1.12)	1.00 (0.91–1.11)	0.98 (0.90–1.06)	1.00 (0.92–1.09)
High (>15)	1.07 (0.96–1.18)	0.96 (0.84–1.08)	0.98 (0.90–1.07)	0.99 (0.89–1.10)
*P-value* ^a^	*0.0008*	*0.1148*	*0.1142*	*0.5287*
*ICC*	*12.6*	*2.1*	*12.3*	*2.1*
Frequency of bus services at “best stop”				
Low (≤ 10)	1.00	1.00	1.00	1.00
Medium low (11–20)	**1.21 (1.10–1.32)**	**1.09 (0.99–1.19)**	**1.19 (1.09–1.30)**	**1.10 (1.01–1.19)**
Medium high (21–40)	**1.43 (1.30–1.57)**	**1.15 (1.04–1.26)**	**1.37 (1.25–1.50)**	**1.16 (1.06–1.27)**
High (>40)	**1.99 (1.77–2.24)**	**1.26 (1.11–1.43)**	**1.62 (1.46–1.81)**	**1.18 (1.05–1.32)**
*P-value* ^a^	*<0.0001*	*<0.0001*	*<0.0001*	0.0142
*ICC*	*7.2*	*1.6*	*8.0*	*1.8*
Bus convenience at nearest stop				
Low (1)	1.00	1.00	1.00	1.00
Medium-low (2)	**1.17 (1.06–1.29)**	**1.12 (1.01–1.25)**	**1.15 (1.04–1.27)**	1.10 (1.00–1.21)
Medium-high (3)	1.07 (0.98–1.16)	1.06 (0.97–1.15)	1.05 (0.97–1.14)	1.05 (0.97–1.41)
High (4)	**1.30 (1.19–1.43)**	**1.19 (1.08–1.32)**	**1.15 (1.05–1.25)**	**1.10 (1.00–1.21)**
*P-value* ^a^	*<0.0001*	*0.0016*	0.0042	0.1591
*ICC*	*11.7*	*2.1*	*12.7*	*2.1*
Bus convenience at “best” stop				
Low (1)	1.00	1.00	1.00	1.00
Medium-low (2)	1.05 (0.98–1.13)	1.01 (0.94–1.08)	1.01 (0.95–1.09)	0.98 (0.91–1.05)
Medium-high (3)	**1.19 (1.10–1.29)**	1.08 (1.00–1.17)	**1.13 (1.05–1.22)**	**1.07 (0.99–1.15)**
High (4)	**1.28 (1.12–1.47)**	1.14 (0.99–1.32)	**1.10 (0.99–1.23)**	**1.06 (0.94–1.19)**
*P-value* ^a^	*<0.0001*	*0.1175*	0.0021	0.0857
*ICC*	*12.3*	*2.0*	*11.8*	*2.1*

**^a^** *P*-value from type III test of the association. **^b^** All models adjusted for neighbourhood confounders population density, median income, street connectivity. Bus routes at nearest stop adjusted for individual confounders distance to nearest bus stop, bus frequency at nearest stop, age, gender and education. Bus frequency at nearest bus stop adjusted for individual confounders distance to nearest bus stop, bus routes at nearest stop, age, gender, education. Bus convenience at nearest stop adjusted for individual confounders bus routes at nearest stop, age, gender and education. Bus frequency at “best” stop and Bus convenience at “best” stop adjusted for individual confounders density of bus stops within 1 km, age, gender and education.

Unique bus routes and transport modes within walking distance showed a positive trend but having a high number of bus routes and three transport modes were not significantly associated with higher odds of meeting the recommendations of physical activity. No significant association was found between bus services at the nearest stop or bus convenience and meeting recommended levels of physical activity.

The ICC in the two empty models showed a noticeable significant between-neighbourhood variation of 13.6% in being an active commuter and 12.7% in meeting recommendations of physical activity. The ICC in the unadjusted models varied from 5.3% to 12.7% and was significantly reduced to between 1.6% and 2.1% in the fully adjusted models.

#### 3.1.4. Subgroup Analysis

For respondents with commute distance ≤10 km, increasing density of bus stops, bus routes within 1 km, frequency of bus services at “best” stop, bus convenience at the nearest stop and TMI at 1 km and 3 km were all positively associated with significantly higher odds of being an active commuter (models not presented). Furthermore, there was a trend for bus convenience at the “best” stop to be related to active commuting. For respondents with commute distances >10 km the associations were insignificant to a large extent. For meeting recommendations of physical activity, the subgroup analysis showed a strong positive association between all density measures and ≥30 min of active commuting per day for those with commute distances of 5–10 km. In those having ≤5 km commute distance, there was a positive trend between the density measures and ≥30 min of active commuting per day. For commute distances >10 km the associations was insignificant.

For women, increasing distance to public transportation was associated with lower odds of being an active commuter and higher density was associated with higher odds of being an active commuter. For men, the associations were insignificant to a large extent and with no clear trend. Only transport modes accessible within 3 km showed a trend towards increasing number of transport modes being associated with significantly higher odds of being an active commuter. The associations for women attenuated in the models of meeting recommended levels of physical activity but remained significant.

For the age group between 30 and 45 the significant associations found in the adjusted models remained. These associations were significant but less pronounced in the age group between 46 and 64. For the age group between 16 and 29, the associations were insignificant to a large extent and with no clear trend for both being an active commuter as well as meeting recommendations of physical activity.

### 3.2. Discussion

The findings of the present study suggest that being an active commuter is associated with proximity to public transportation, number of bus routes, bus service frequency and accessible transport modes within walking or cycling distance. Public transportation characteristics that facilitate active commuting are thus complex and need to be better modeled and described than by distance measures alone.

This study strengthens the evidence on the association between access to public transportation and active commuting and advances previous research by introducing different density measures not only based on access but also on transit services and accessible transport modes. The study highlights the importance of considering not only the nearest stop but also alternative services to describe access to public transportation. While no significant association was found for number of routes and service frequency at the nearest stop, positive associations were found between bus service frequency and being an active commuter at the “best” stop. This suggests two very different conclusions about the association between public transportation and active commuting. In urban or suburban areas the “best” stop might be located close to the nearest stop; therefore, it is important to include other stops than nearest stop in measures of local public transportation facilities [41]. Having access to more transport mode choices than a bus within walking or cycling distance also had a positive effect on being an active commuter. One explanation for the non-significant associations with the nearest stop might be that the variation in the measures at the nearest stop is too low to reveal an association. The association with the bus frequency has only been investigated in relation to active commuting in few other studies [15,21,42]. Dalton *et al.* [15] found that medium (tertiles) and low bus frequencies were significantly associated with lower odds of using public transportation compared to having a high frequency. Kamada *et al.* [21] did not find a significant association, but their sample size was small and therefore had very low statistical power.

In accordance with other studies [15,18,19,20,21], negative associations were found between the distance to the nearest stop or station and being an active commuter as well as with meeting recommendations of physical activity. The results suggests that shorter walking distances to a bus stop supports active commuting, whereas the attractiveness of the other public transportation modes (metro, trains and s-trains) diminishes with access distance. This is in line with other studies showing that people will walk further to trains than to buses [42,43]. Due to the large study area many respondents have very long distances (>3 km) to the train, S-train and metro stations. This clearly attenuates the associations for these three transport modes. Locally, the three transport modes are very important for commuting by public transportation in the region with direct and fast services to the main city centres.

The positive associations found between the different density measures and being an active commuter is supported by other studies findings [22,24,25,26]. The alternative density measures, the unique bus routes within 1 km and number of transport modes measures the effect of having additional services within walking distance and show strong associations with active commuting. The outcomes for the density measures reveal the importance of both easy access to public transportation and to different transport modes and routes that enable more destinations to be reached. The positive associations found may not only reflect a higher use of public transportation, walking and cycling in areas of high density, but also restrictions for car use such as lack of parking space and traffic congestion [39] and better connected street networks that allow more direct travelling and the presence of cycle lanes that facilitate active commuting.

In the present study, having a high frequency and a short distance to the nearest stop are associated with significantly higher odds of being active compared to having longer distances and low frequency. This was not significant for the “best” stop convenience measure or in association to meeting the recommendations of physical activity. Kamada *et al.* [21] did not find a significant association between their convenience measure and active commuting, but their results showed the same positive trend that higher convenience was associated with higher odds of active commuting. It is highly questionable, however, whether the two studies are comparable as Kamada *et al.* [21] investigated women living in a rural setting in Unnan City, Japan, with a generally low public transportation service level.

The findings suggest that better access to public transportation through shorter distances between stops (higher access coverage) and higher diversity in bus routes and transport modes is associated with active commuting. From a planning perspective, it is less costly to change the service frequency than to make infrastructural changes to promote active commuting. Further studies could evaluate what level of service frequency is the most attractive to commuters. Studies have found that commuters are willing to walk further to some transit services than others [43]. Providing fewer but better served stops could potentially promote active commuting through more walking and cycling to stops. A better service level may have a larger impact in the rural areas characterized by low public transportation access coverage.

The finding of a significant interaction with commute distance is in line with previously presented results; indicating, that distance to work is a strong predictor of travel mode choice when commuting [15,17]. When the commuting distance is > 10 km the number of commuters who cycle all the way to work decreases markedly and car-based commuting becomes dominant [40]. This is also evident from the results of this study where car-based commuting is likely to be the main reason why the associations were weaker for the respondents residing far (>10 km) from work or study.

Women’s commute travel choices seem to be more influenced by access to public transportation than men. The associations found in the full model remain significant and in the same magnitude for women, but for men these associations become insignificant. Laverty *et al.* [4] finds a similar association with travel mode, indicating that women are more likely to walk or use public transportation when commuting than men. Men’s travel choice may be more influenced by car ownership. However, data on car ownership were not available in the present study.

The 16 to 29 year olds are to a large extent walking or cycling in combination with using public transportation which may explain the non-significant associations between the access measures and being an active commuter in this age group. The travel choice in the 30 to 45 year old group seems to be much more influenced by access to public transportation and a higher access and service level result in higher odds of being an active commuter and meeting recommended levels of physical activity. The associations become less pronounced for the 46 to 64 year olds. This may be the result of more car-based commuting and possibly also caused by less cycling or walking due to functional decline with age.

One of the challenges for future transport planning is to create solutions that enable time-competing multimodal trips for those with longer commute distances, thereby incorporating active transportation into otherwise car dominant commuting. Many initiatives have already been implemented to increase multimodal travelling in Denmark. It is now possible to get your bicycle on the S-train, some busses and trains. This enhances the availability of public transportation both in the access and the egress stages thereby reducing the weakest part of a multimodal trip [31]. Shared bicycles have been introduced in many European cities and a study from Helsinki, Finland [44] show that bicycle sharing could reduce public transportation travel time in the study area by more than 10%. Better walk paths between adjacent, but not connected, public transport stops have also been found to improve transfer and thereby reduce travel time [45]. Furthermore Copenhagen and adjacent municipalities are introducing super cycle highways from the suburban areas to Copenhagen City centre with only a few stops with resulting reduction in travel time [46]. Infrastructural changes should be supported by restrictions on car parking which has been shown to reduce car-based commuting [40]. Promotion of public transportation through more direct routes and reduction of fare prices may also have a positive impact [47].

The potential for active commuting to provide health benefits is evident from a number of studies on total physical activity. These studies find that commuting solely by active modes, or active commuting in combination with public transportation or car, was significantly more active than only using motorized modes of transport [11]. Transit users has been found to have higher daily levels of total physical activity, but did not differ in non-transit related walking or non-walking physical activity [48]. The current study showed a high level of respondents benefitting from active commuting and meeting recommended levels of physical from active commuting alone. In contrast, a recent study performed in the Capital Region of Denmark [49] shows that while the number of bicycle trips rose in the Copenhagen City Center and suburban areas from 2007 to 2012, a decrease was found in the other municipalities. This stresses the need for addressing strategies to support those with lower access to public transportation.

#### Strengths and Limitations

This study has several strengths. The individual GIS-based objective measures for distance from home address to public transportation, calculated using network analysis and the inclusion of transport service characteristics, provided a sound setting for studying the association between access to public transportation and active commuting. The study tested a wide range of new objective density measures and the inclusion of the “best” stop in the analysis enabled a discussion to take place about how well conclusions based on nearest stop capture the significance of public transportation to active commuting. The large study population selected from one of the largest health surveys in the world and the individual register-based socioeconomic data provide a unique study base. Estimates of the ICC showed a clear amount of variation between the neighbourhoods on the outcome variable. The neighbourhood effect was accounted for in the 3-step multilevel model and significant reduction in the variation among neighbourhoods was observed.

The main limitations of the study are the self-reported daily active commuting which may be subject to information bias. Respondents might have reported daily active commuting even though they had only taken the bus a few times a month, which would have made the share of active commuters too high. The survey is cross-sectional in design so it is not possible to conclude on causality and self-selection may lead to an apparent association between active travel and transit facilities [50]. Adjusting for individual education in combination with employment (selected population) and neighbourhood SES, met some of the limitations regarding self-selection [51]. The high proportion of respondents reporting active commuting in this study (72.9%) is substantially higher than in other studies. Results may therefore not be generalizable to other countries or cities where active commuting is not as common. However, the observed associations are quite similar to other studies, which indicate that the findings may be comparable. The Health Survey had a response rate of 52.3%. The implication of this response rate was tested in a non-response analysis. The analysis showed that the response rate was highest among women, middle-aged individuals and individuals of higher socio-economic position and lowest among men, young and elderly individuals and individuals of lower socio-economic position. Accordingly a number of statistic weights have been calculated by Statistics Denmark to adjust for the non-response on municipality level. The weights have not been applied to the individual data in this study because the difference between including and not including the weights in the regression analysis gave no significant difference in the estimates. This may be due to the fact that this study analyses individuals and the weights are based on the municipality population.

The active commuting information is restricted to time spent walking or cycling to work or study, and it does not refer to time spent in usage of public transportation or car. This restricts the analysis to looking at active commuting and not the individual choice of travel mode. It is not possible to examine the association between use of public transportation and active commuting in this study, but only the association between access to public transportation and active commuting in general. Further studies would benefit from including information on commute mode in order to investigate the association between different features of the built environment and different commute travel behaviours. The Health Survey 2013 has been expanded and includes this information. The outcome variable also makes it difficult to separate non-active commuters and active commuters. The 5 min cut-off value was chosen under the assumption that car-based commuters report none or only a few minutes of walking and cycling to work or study. 73.8% report any active commuting, 72.9% report 5 min or more, 69.6% report 10 min or more and 50.6% report 30 min or more. The associations are thus strong with low sensitivity to the cut-off value. Due to the high bus coverage especially in the Copenhagen metropolitan area, 5 min were chosen as cut-off value to ensure that short trips walking to and from transit were captured in the analysis.

Whether the services at nearest or “best” stop was able to transport the respondents to work or study is unknown. The distance measures are based on network distances along the road network. Walkers and cyclists are likely to follow cut-throughs such as crossing green areas, taking alternative paths or street crossings not included in the road network. The shortest path distances to public transportation used in this analysis may thus overestimate the “real” walking or cycling distances.

A number of confounders identified in other studies were not included in this study. Car ownership is often a strong predictor in analyses of travel mode choice [52]. It was not included in this study as it was not the aim to investigate how car ownership affects active commuting. Health measures such as general health state, disability and chronic diseases may affect travel choice and it would be good to include those in further analyses.

## 4. Conclusions

The results of the study show that easy access to public transportation and high frequency transport services have the potential to promote active commuting. The results suggest that active commuting is associated with the proximity and density of public transportation stops; however, transport service characteristics such as number of routes, service frequency and accessible transport modes within walking or cycling distance are also important factors associated with active commuting by public transportation. Future research should include an evaluation of public transportation service level and characteristics at all stops within walking distance and it should incorporate travel choices to gain a better understanding of the driving forces behind active commuting. Commute distance is seen to be limiting active commuting. Planning should promote easier incorporation of active transportation into otherwise car dominant commuting through initiatives such as bicycle sharing systems and better infrastructure for active transportation modes. From a public health perspective promotion of active commuting has the potential to provide health enhancing physical activity to commuters.
